# Quantifying transmission and immunity dynamics of multiple SARS-CoV-2 variants using models and epidemic data from a highly populated area

**DOI:** 10.1371/journal.pone.0327817

**Published:** 2025-07-16

**Authors:** Monica S. Shah, Jiyoung Lee, Laura W. Pomeroy

**Affiliations:** 1 Environmental Sciences Graduate Program, The Ohio State University, Columbus, Ohio, United States of America; 2 Division of Environmental Health Sciences, College of Public Health, The Ohio State University, Columbus, Ohio, United States of America; 3 Department of Food Sciences and Technology, College of Food, Agricultural, and Environmental Sciences, The Ohio State University, Columbus, Ohio, United States of America; 4 Infectious Diseases Institute, The Ohio State University, Columbus, Ohio, United States of America; 5 Translational Data Analytics Institute, The Ohio State University, Columbus, Ohio, United States of America; Shahid Beheshti University of Medical Sciences School of Medicine, ISLAMIC REPUBLIC OF IRAN

## Abstract

Identifying temporal patterns in dynamics of acute, immunizing infectious diseases informs our understanding of transmission, epidemic prediction, and disease control. However, for emerging pathogens like SARS-CoV-2, temporal dynamics remain underinformed, even though COVID-19 cases varied greatly over time. Using nested compartmental models, we quantified transmission and immune dynamics in part of Columbus, the capital city of the state of Ohio, United States (US). We parameterized models using state-reported COVID-19 case counts and wastewater-based surveillance (WWS) for SARS-CoV-2. We used the models to reconstruct transmission and the rate of waning immunity in three distinct pandemic phases from April 2020 to August 2022. On average, transmission rates were lowest for the ancestral strain and highest for the Omicron variant. Transmission did not display consistent seasonal changes but did vary through time in ways that might have been influenced by host behavior or viral strain switching. Our findings also indicate that vaccine-induced and infection-induced SARS-CoV-2 immunity wane at similar rates. Gaining a better understanding of population-level transmission and immune dynamics following the emergence of a novel pathogen can inform future public health interventions including vaccine schedules.

## Introduction

Dynamics of acute, immunizing infectious diseases are often driven by the aggregation of children during school terms, birth rates, and vaccination [1–6]. However, for emerging pathogens to which the entire population is susceptible and no vaccinations are yet available, temporal dynamics remain undercharacterized; this added uncertainty when designing and implementing disease control during the recent COVID-19 pandemic. SARS-CoV-2, the coronavirus that causes COVID-19, was first detected in December 2019 [7]. The virus caused an enormous amount of disease, and by December 2024, over 777 million cases were reported globally [8]. Case counts varied greatly over time and were likely shaped by changes in human behavior, the virus itself, and host immune status, including vaccination. Behavior aimed at preventing transmission—face masks, online school, large gathering cancellations—varied over time. In addition, the dominant circulating SARS-CoV-2 strain changed over time, with variants displaying differing transmissibility [9,10] and different degrees of cross-immunity [9,11–13]. Vaccines that were nonexistent at the start of the COVID-19 pandemic became widely available in the US during the winter of 2020, and boosters became available the following fall. Identifying how changing human behavior, viral strain switching, and host immunity interacted to shape infection dynamics is essential to better understand and predict epidemics and can inform the design of control for SARS-CoV-2 and other acute, immunizing pathogens.

One approach to quantifying transmission dynamics and immune status is to use models calibrated with host and disease data. Mathematical models that describe infectious disease dynamics have a rich tradition of quantifying dynamics of acute, immunizing pathogens [14–16]. Statistically fitting models to case count data can quantify parameters that represent transmission and immunity. Models can also be designed to represent competing hypotheses about transmission and immunity, and the best-fit model can be statistically selected, indicating which hypothesis has the most support [17,18]. Models parameterized with case count data have quantified transmission and rates of waning immunity across a range of host-pathogen systems [17,19–21].

Calibrating models of infectious disease dynamics with case count data can be informative, but it requires cases to be reported and tabulated over time, which was difficult during the earliest phases of the COVID-19 pandemic. For an individual infected with SARS-CoV-2 to be counted as a COVID-19 case in data reported by the state of Ohio, the infected individual must have successfully completed multiple steps. First, sick individuals must have self-identified. But, with approximately 40% of COVID-19 cases presenting asymptomatically [22], individuals without symptoms may have been unaware of their disease status. Second, individuals must have obtained a diagnostic COVID-19 test. But, true or perceived lack of access to tests, especially early in the pandemic and during surges in case counts [23], could have led to decreased testing. Third, if individuals tested outside of the office of a medical provider, they must have reported results to a public health entity. If the positive test result was not reported in a specific manner—often with additional metadata like name, residence, and age that some databases required—positive cases might not be counted. For these reasons and other difficulties, official reported case count data provides insight into case counts, but they are extremely likely to be underestimated [24].

One potential way to increase accuracy of disease data might be to augment them with wastewater-based surveillance (WWS) data. WWS has a history of informing trends in case counts of polio virus [25–27], hepatitis A virus [28,29], and most recently, influenza A virus [30]. Because most individuals infected with SARS-CoV-2 shed viral RNA via their feces [31–33], the quantity of viral nucleocapsid (N) gene copies in wastewater entering a treatment plant correlates with reported case counts [34–36]. WWS data may capture infected individuals regardless of symptoms [37,38] and testing behaviors [39], which might help alleviate underreporting inherent in reported case data. Alternatively, WWS data might introduce other sources of uncertainty due to individual variability in the amount of viral RNA shed or rain water dilution of samples [40,41]. How to integrate WWS data with case count data to inform dynamics and drivers of acute, immunizing infectious diseases remains an area of current research.

Our goal is to understand how SARS-CoV-2 transmission and immune status varied over time during the early phases of the COVID-19 pandemic using both case count and WWS data. Specifically, during the first two years of the pandemic (April 5, 2020 through August 27, 2022), we seek to (i) quantify temporal variation in transmission and (ii) quantify the per capita rates at which infection-induced and vaccine-induced immunity waned. For these reasons, we model SARS-CoV-2 transmission and immunity in the northwest region of Franklin County, Ohio, USA, which includes the campus of Ohio State University, part of the city of Columbus, Ohio, and some surrounding suburbs. This research identifies and quantifies variation in population-level transmission and immunity following the introduction of a novel, emerging pathogen, which may inform future epidemics and help design effective control measures.

## Materials and methods

### Study population and data

**Fig 1:**
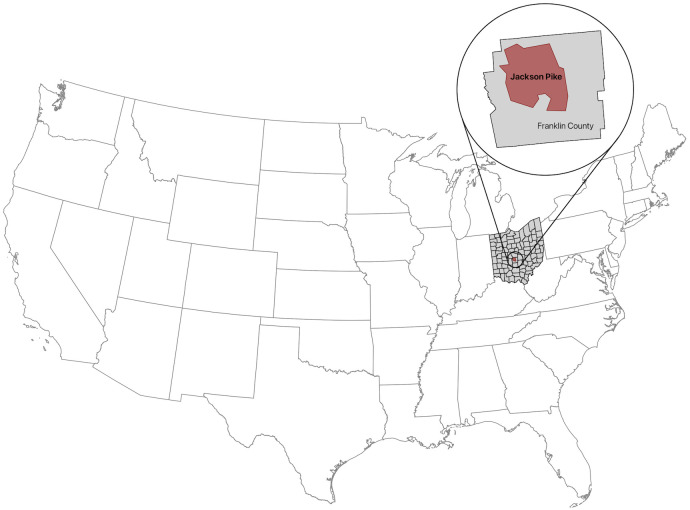
Location of the Jackson Pike sewershed in northwest Franklin County, Ohio, USA. The study population is defined as all individuals with residences within the Jackson Pike sewershed (outlined in red). The Jackson Pike sewershed is in Franklin County, Ohio, USA and includes three municipalities: part of the city of Columbus, OH; the northwestern suburbs of Columbus, OH; and Ohio State University. The map was prepared using the Cartographic Boundary Files dataset from the U.S. Census Bureau [44].

Our study included all individuals who reside within the Jackson Pike sewershed in northwest Franklin County, Ohio, USA (Fig 1). The sewershed includes three municipalities: part of the city of Columbus, a large metropolitan area with a high population density; the northwestern suburbs of Columbus, which also have a high population density; and Ohio State University. Approximately 645,940 individuals live in the sewershed [34], and it serves 49% of Franklin County residents. We obtained data on live births and data on mortality not attributable to COVID-19 from the Ohio Department of Health (ODH) Public Health Information Warehouse [42]. We obtained data on mortality attributable to COVID-19 from the ODH COVID-19 dashboard [43]. Birth and death data were only available at the county-level, so we attributed a proportionally scaled amount (49%) of births and deaths to our study population. Please see the S1 File for more details.

### Disease and vaccination data

We obtained two sources of disease data. First, we obtained daily COVID-19 case count data among residents in the sewershed from the ODH COVID-19 Dashboard’s Coronavirus Wastewater Monitoring Network, which started on January 27, 2020 [45]. Second, we obtained twice-weekly WWS data from the Ohio Coronavirus Wastewater Monitoring Network [45,46] starting on July 19, 2020. Samples were taken from wastewater entering the treatment plant (influents) on Sundays and Tuesdays [34,45]. A previous study by Ai *et al.* (2021) demonstrated that the concentration of the N2 gene type of SARS-CoV-2 is highly correlated with case numbers in the Jackson Pike sewershed [34]. To integrate both data sources into the same analysis, we converted both data sets to weekly case counts. To convert the daily COVID-19 case count data, we aggregated reported daily values by weeks, starting on Sundays. To convert the WWS data, we used the simplified equation in McMahan *et al.* (2021) to estimate daily prevalence as the total number of infected individuals in a 24-hour period [47]. Because COVID-19 has an infectious period of 7 days [48], we averaged the two values calculated each week to obtain weekly incidence (S1 File). We compared weekly state-reported COVID-19 case count data and weekly counts of infected individuals estimated by WWS data using Spearman’s correlation coefficients. We used the weekly maximum value from comparing the reported case count and WWS-derived incidence to parameterize our models.

We obtained vaccination data from the ODH COVID-19 dashboard [43]. The SARS-CoV-2 vaccine was offered as both a single dose and multiple dose series depending on the manufacturer; ODH reported vaccination when recipients completed the single-dose vaccine or all doses of the multiple-dose vaccine (and booster doses). Daily vaccination data was reported by the recipient’s county of residence. We scaled this county-level data to our study population in the same way that we scaled the birth and death data, assuming 49% lived in the sewershed, and aggregated daily values by weeks starting on Sundays. We calculated the proportion of sewershed residents who were vaccinated each week.

### Models of transmission and immunity

**Fig 2:**
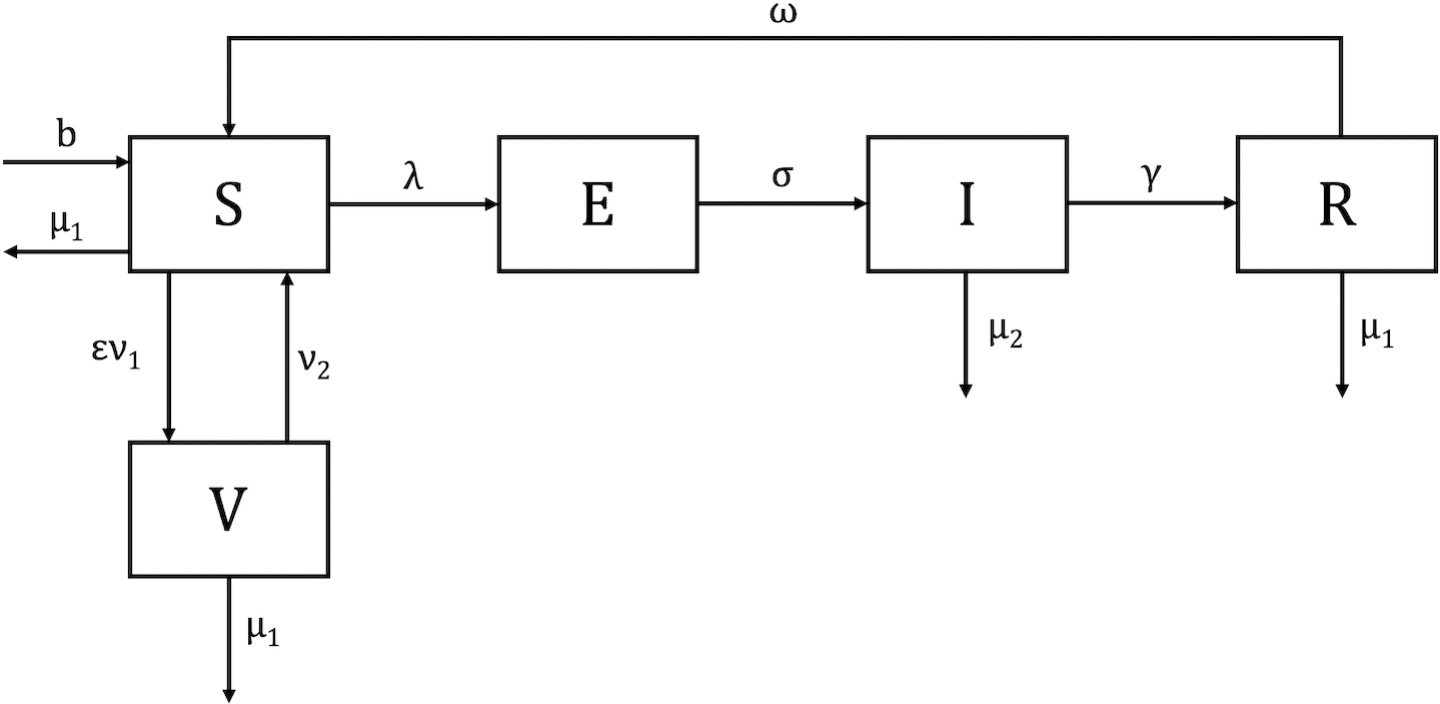
Model of SARS-COV-2 transmission and immunity. Individuals in the susceptible (S) category can be infected. Births enter the susceptible category at quantity b, and susceptible individuals die at rate μ_1_. Susceptible individuals become infected at rate λ and enter the Exposed (E) category or become vaccinated at a rate ν_1_ multiplied by the proportion that mount protection ε and enter the Vaccinated (V) category. Exposed individuals progress to the Infectious (I) category at rate σ. Death rates are not considered for exposed individuals as only one week is spent in this category. Infected individuals recover at rate γ and enter the Recovered (R) category or die from infection at rate μ_2_. Recovered individuals experience infection-induced immunity waning at rate ω or die at rate μ_1_. Vaccinated individuals experience vaccine-induced immunity waning at rate ν_2_ or die at rate μ_1_. As applicable in each phase, we test the following hypotheses: (a) neither infection-induced immunity nor vaccine induced immunity wanes (ω = 0, ν_2_ = 0), (b) infection-induced immunity wanes but vaccine-induced immunity does not wane (ω > 0, ν_2_ = 0), and (c) both infection-induced immunity and vaccine induced immunity wane (ω > 0, ν_2_ > 0).

Because we aimed to quantify SARS-CoV-2 transmission over time and test multiple hypotheses about if and how protection against reinfection is lost, we used a susceptible, exposed, infectious, recovered, susceptible (SEIRS) model to represent infection dynamics before vaccination was available starting on April 5, 2020. We used a susceptible, exposed, infectious, recovered, vaccinated, susceptible (SEIRVS) model to represent infection after vaccination was available, starting on January 3, 2021 [49,50]. These models categorize individuals by their disease status (Fig 2).

Susceptible individuals (S) do not have protective immunity against the current, circulating SARS-CoV-2 variant and can be infected. Upon infection, a susceptible individual moves into the exposed category (E), defined as those who are infected with the virus but not yet infectious. An exposed individual becomes infectious after one week [51] and moves into the infectious category (I). Infectious individuals mount protective immunity and enter the recovered category (R) after one week as they are no longer infectious [48].

Let E_t_ represent the number of exposed individuals at weekly timestep t. The of number newly infected but not yet infectious individuals at timestep t + 1, can be calculated as


$$E_{t+1}=E_{t}+\lambda_{t}S_{t}-\sigma E_{t},$$ (1)

where S_t_ represents the number of susceptible individuals, λ_t_ represents the force of infection, and σ represents rate at which individuals move from the exposed category (E) to the infectious category (I) (S1 Table in S1 File). We assumed SARS-CoV-2 was transmitted in a density-dependent manner and calculated force of infection as


$$\lambda_{t}=\beta_{t}I_{t},$$ (2)

where β_t_ represents the weekly transmission coefficient, or the probability that a contact between a susceptible and infectious individual will lead to disease transmission multiplied by a constant [52] and I_t_ is the number of infectious individuals during timestep t.

Let R_t_ represent the number of recovered individuals at weekly timestep t. The number of recovered individuals at the following timestep t + 1 can be calculated as


$$R_{t+1}=R_{t}+\gamma I_{t}-\omega R_{t}-\mu_{1,t}R_{t},$$ (3)

where γ is the per capita recovery rate, ω is the per capita rate at which infection-induced immunity wanes, and μ_1,t_ is the proportion of individuals dying from reasons other than COVID-19 during timestep t (S1 Table in S1 File).

Let V_t_ represent the number of vaccinated individuals at weekly timestep t. The number of vaccinated individuals at the following timestep t + 1 can be calculated as


$${\;\;\;\;\;\;\;\;\;\;\;\;\;\;\;\;\;\;\;\;\;\;V}_{t+1}=V_{t}+{\varepsilon \nu }_{1,t}S_{t}-\nu_{2}V_{t}-\mu_{1,t}V_{t},$$ (4)

where ν_1,t_ is the proportion of people receiving the vaccine during timestep t, ε is the proportion of individuals who mount immunity from the vaccine, μ_1,t_ is the proportion dying from reasons other than COVID-19 during timestep t, and ν_2 _is the per capita rate at which vaccine-induced immunity is lost. Because the vaccine may be imperfect in protecting against infection, we assumed that 90% of people receiving the vaccine each week mount protective immunity and set ε as 0.90 (S1 File).

### Model selection, implementation, and computing

We used the models to simultaneously quantify SARS-CoV-2 transmission over time and test competing hypotheses about population-level immunity during different phases of the pandemic. Before vaccines became available, we tested the following two hypotheses: (a) infection-induced immunity wanes (ω > 0) and (b) infection-induced immunity does not wane (ω = 0). After vaccines became available, we tested three hypotheses: (a) neither infection-induced immunity nor vaccine-induced immunity wanes (ω = 0, ν_2_ = 0) (b) infection-induced immunity wanes but vaccine-induced immunity does not wane (ω > 0, ν_2_ = 0), and (c) both infection-induced immunity and vaccine-induced immunity wane (ω > 0, ν_2_ > 0). We fit models (equations 1–4) representing each hypothesis to disease data using Poisson likelihoods using the *optim* function in R. We selected the best-fit model from each pandemic phase using Akaike Information Criterion (AIC) value [53] and used the best-fit model to quantify values for three parameters (monthly transmission coefficient (β_t_), per capita rate of infection-induced immunity waning (ω), and per capita rate of vaccine-induced immunity waning (ν_2_), please see S1 File for additional details).

We fit models to timeseries of disease data separately for three different pandemic phases: (i) the period from April 5, 2020 to January 2, 2021, when the original or ancestral SARS-CoV-2 variant was circulating and vaccines were not yet available; (ii) the period from January 3, 2021 to December 4, 2021 when multiple SARS-CoV-2 variants of concern (Alpha, Beta, Gamma, and Delta) were circulating and the monovalent vaccine was available; and (iii) the period from December 5, 2021 to August 27, 2022, when the Omicron SARS-CoV-2 variant was circulating and the monovalent vaccine was available. We identified dominant SARS-CoV-2 strains using the CDC’s SARS-CoV-2 Variant Genomic Surveillance data for Health and Human Service (HHS) Region 5, which includes Ohio [54]. These data were first reported in January 2021; therefore, we identified earlier SARS-CoV-2 as the original or ancestral strain. We set the initial count of infectious individuals (I_0_) as the case count on April 5, 2020, the initial count of exposed individuals (E_0_) as in the case count one week later, the initial count of recovered individuals (R_0_) as the cumulative case count before April 5, 2020, and the initial count of susceptible individuals (S_0_) considering the total population size (N_0_) assuming that N_0_ = S_0_ + E_0_ + I_0_ + R_0_. For the second and third pandemic phases, we set the initial conditions as the values from the last week of simulations of the best-fit model from the previous phase.

All calculations, data analysis, model building, model selection, parameter estimation, and graphing were performed in R version 4.2.2 [55] using RStudio [56].

## Results

### Disease data

**Fig 3:**
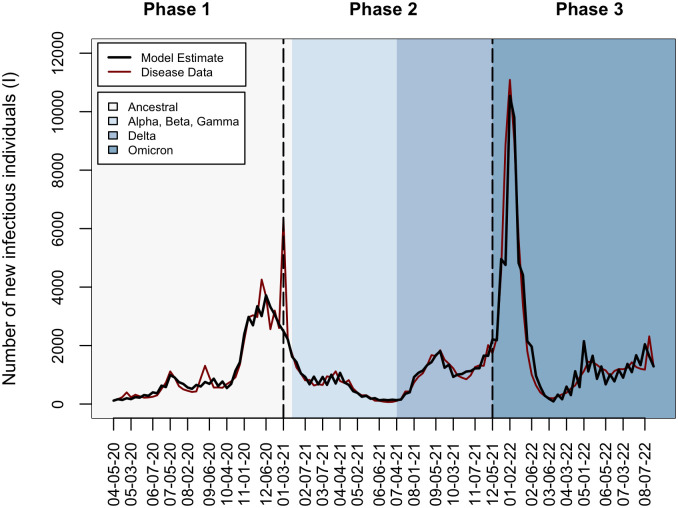
Model output. Incidence estimated by the best-fit model for each phase (black line) plotted against the maximum value of the case count or WWS incidence data each week (red line). Dashed vertical lines represent the start of each pandemic phase, and background colors indicate the dominant circulating strain.

Between April 5, 2020 and August 27, 2022, 157,872 new COVID-19 cases in this sewershed were reported to the ODH. Between July 19, 2020 and August 27, 2022, 100,703 cases were estimated by the WWS data. The counts of new cases per week generated by the two surveillance methods were highly correlated throughout this period (Spearman’s rank correlation = 0.75; p-value < 2.2 × 10^−16^). Correlation differed by pandemic phase: the first period (April 5, 2020 to January 2, 2021) showed the highest correlation (Spearman’s rank correlation = 0.87; p-value = 2.5 × 10^−6^), followed by the third period (December 5, 2021 to August 27, 2022; Spearman’s rank correlation = 0.78; p-value = 2.67 × 10^−7^). The second period (January 3, 2021 to December 4. 2021) had the lowest correlation (Spearman’s rank correlation = 0.57; p-value = 2.61 × 10^−5^). Reported cases were higher than the wastewater-derived case count estimates for most (78.9%) of the weeks in our study. The weekly maximum value from comparing the reported case count and WWS-derived incidence yielded 168,329 cases over the study period. (Fig 3; red line).

### Model selection and parameter estimation

**Fig 4:**
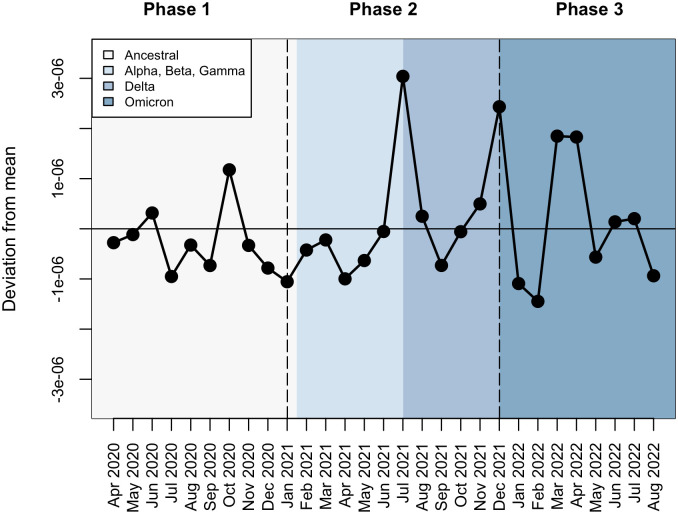
Monthly variation in transmission. Transmission coefficients each month were calculated from the best fit model of each pandemic phase. Monthly transmission coefficients were averaged throughout the study period to find a mean value (horizontal line). Black dots represent each month’s deviation from the overall mean transmission coefficient. Horizontal dashed lines delineate each phase of the pandemic, and the background color represents the circulating variant. Transmission did not display seasonality but did vary over time.

We fit models that represented competing hypotheses on the nature of SARS-CoV-2 immunity to disease data independently for each of the three pandemic phases (Fig 2). Using the model that fit the data better (i.e., had the lowest AIC value) for each pandemic phase, we quantified monthly variations in the transmission coefficient. Average monthly transmission was lowest when the original strains of SARS-CoV-2 were dominant, mid-range when multiple variants of concern were circulating, and highest when Omicron was the dominant circulating variant (Table 1). Transmission did not display consistent seasonality during the first two years of the pandemic (Fig 4). The highest transmission rates occurred during October 2020, July 2021, December 2021, and March 2022 through April 2022 (Fig 4).

**Table 1:** Model comparison.

Dates	Epidemic characteristics	Models	AIC value	Average transmission coefficient (β)	Per capita rate of infection-induced immunity waning (ω)	Per capita rate of vaccine-induced immunity waning (ν_2_)
April 5, 2020 to January 2, 2021	Ancestral SARS-CoV-2 strain dominant; no vaccine available.	**No loss of infection-induced immunity**	**2589.64**	**1.91x10** ^**-6**^	**–**	**–**
		Loss of infection-induced immunity	2596.38	1.92x10^-6^	0.066 wks^-1^ (1/15.1 weeks)	–
January 3, 2021 to December 4, 2021	Multiple SARS-CoV-2 variants of concern including Alpha, Beta, Gamma, and Delta dominant; monovalent^1^ vaccine available.	No immunity loss (infection-induced nor vaccine-induced)	6046.30	2.85x10^-6^	–	–
		Loss of infection-induced immunity only	6065.10	2.87x10^-6^	0.0014 wks^-1^ (1/725 weeks)	–
		**Infection-induced and vaccine-induced immunity loss**	**6012.50**	**2.10x10** ^**-6**^	**0.052 wks** ^**-1**^ **(1/19.4 weeks)**	**0.051 wks** ^**-1**^ **(1/19.7 weeks)**
December 5, 2021 to August 27, 2022	Omicron SARS-CoV-2 variant dominant; monovalent^1^ vaccine available.	No immunity loss (infection-induced nor vaccine-induced)	8249.37	3.11x10^-6^	–	–
		Loss of infection-induced immunity only	7991.56	2.85 x10^-6^	0.038 wks^-1^ (1/26.3 weeks)	–
		**Infection-induced and vaccine-induced immunity loss**	**7903.88**	**2.40x10** ^**-6**^	**0.037 wks** ^**-1**^ **(1/27.3 weeks)**	**0.033 wks** ^**-1**^ **(1/30.0 weeks)**

^1^Early monovalent COVID-19 vaccines from dates were based on the ancestral SARS-CoV-2 viral strain.

We estimated the duration of infection-induced (ω) and vaccine-induced immunity (ν_2_) by statistically estimating the per capita rate at which each form of immunity wanes. During the 8-month period when the original strains of SARS-CoV-2 were dominant and vaccines were not yet available, we found no loss of infection-induced immunity (Table 1). During the 11-month period when multiple variants of concern were circulating (Alpha, Beta, Gamma, and Delta) and vaccines first became available, we found a loss of both infection-induced and vaccine-induced immunity. Infection-induced immunity and vaccine-induced immunity waned at similar rates (0.052 wks^-1^ and 0.051 wks^-1^, respectively; Table 1). During the 8-month period when Omicron was the dominant variant and the vaccine was available, we found a loss of both infection-induced and vaccine-induced immunity (Table 1). Again, infection-induced immunity loss and vaccine-induced immunity loss occurred and similar rates (0.037 wks^-1^ and 0.033 wks^-1^, respectively; Table 1). But, overall, immunity lasted longer than the previous phase.

## Discussion

The objective of this study was to reconstruct transmission during the first two years of the COVID-19 pandemic to quantify time-varying transmission and characterize population immunity changes. On average, monthly transmission was the highest during the time when Omicron was the dominant circulating variant. Transmission did not display consistent seasonal patterns, and the timing of transmission peaks varied by year. We found no support of immunity loss in 2020, but from 2021 onward, we found support of both infection-induced immunity loss and vaccine-induced immunity loss at the population level.

Our study population was unique as it included students, faculty, and staff of Ohio State University. This is a large university with a highly robust testing program, administering a total of close to 850,000 COVID-19 tests to individuals with and without symptoms during this study period [57]. Given that over 10% of the study population attend the university and even more individuals are employees [58], this has interesting implications for our results. First, this large volume of routine testing could explain why incidence estimated from WWS did not consistently estimate more cases than reported, though this could also be attributed to the inherent uncertainty in deriving the number infected from SARS-CoV-2 gene copy data. The estimation of wastewater-derived case counts was highly correlated with state-reported cases, though this association slightly decreased in magnitude as the pandemic progressed. The decreasing correlation could be caused by variant dependent changes in the maximum gene copies shed per grams feces [59] or by geographical differences in where infected individuals contributed to WWS versus case count data as movement restrictions were lifted over time. Second, because we fit models to disease data that represented cases found during routine testing, symptomatic individuals, and those that shed into the wastewater system, the data likely captured positive cases from individuals both with and without symptoms. Therefore, our models considered vaccinated and recovered individuals to have immunity that protects against both symptomatic and asymptomatic infection and our findings indicate that both vaccine-induced and infection-induced immunity wane.

Notably, transmission did not display seasonality, despite this phenomenon being common with other respiratory-borne pathogens [60]. A potential explanation is the variations in behavior during the first two years following the widespread emergence of a novel virus. For example, the presence of control measures, such as social distancing and masking policies, were in place at the start of our study but decreased drastically as the pandemic progressed. Four major peaks in transmission occurred: in October 2020, July 2021, December 2021, and March 2022. The October 2020 and March 2022 peaks may be explained by increased contact through students returning to school after summer and spring break. Given the large number of university students represented in the data and that around 23% of Franklin County is under the age of 18 [61], these increases may be school-term driven, as observed in other directly transmitted human diseases such as influenza [62] and measles [1]. The large peak in July 2021 coincided with the emergence of the Delta variant as the dominant circulating strain; this peak could be driven by the increased transmissibility of the variant or population behaviors, such as lifted mask mandates and increased social contact over the summer, that increased transmission [63] On average, transmission was the highest in 2022 when Omicron was dominant; this aligns with findings of increased transmissibility of the variant [9,12]. Investigating age-structured contact patterns may help better understand the relationship between school cycles and SARS-CoV-2 transmission and elucidate other potential drivers of transmission variation.

We found that infection-induced immunity did not wane during the 38 weeks when the ancestral SARS-CoV-2 strain was dominant and vaccines were not yet available; however, both infection-induced and vaccine-induced immunity waned when other variants of concern (Alpha, Beta, Gamma, Delta) and Omicron circulated. Our findings about long-lasting immunity to the ancestral strain and waning infection- and vaccine-induced immunity while Omicron circulated agree with previous epidemiological studies [64]. Our findings about waning infection- and vaccine-induced immunity to other variants of concern (Alpha, Beta, Gamma, Delta) indicated shorter duration of immunity than that of other epidemiological studies [64]. This may be because our duration estimates of infection-induced immunity represent minimum values: anecdotal information suggests that more infections in our study site may have occurred in January 2020 – not in March or April 2020 like data indicate – and our duration of immunity estimates are based on time to re-susceptibility rather than time to re-infection. While partitioning time into strain-specific eras was necessary to quantify strain-specific transmission, it might obscure our findings about the duration of immunity. For example, immunity conferred to ancestral strains might last longer than 40 weeks [64] before being lost, which would correspond to a loss of immunity at our study site when other variants of concern or Omicron circulated. Additionally, immune-specific assays measuring antibody titers post infection show a decrease around 2–4 months after symptom onset, but remain detectable for at least 6 months post infection [65,66]; our findings estimate a slightly longer duration, potentially because we model infection regardless of symptoms, but are similar to this range. Overall, our results align with previous studies indicating that vaccine-induced and infection-induced SARS-CoV-2 immunity can be lost and that vaccine-induced and infection-induced immunity wane at similar rates. This demonstrates that analyses conducted at multiple scales, from our population-wide modeling to previous within host antibody measurements, corroborate that immunity to SARS-CoV-2 is on the magnitude of months long.

Our study had inherent limitations, due to the data and the assumptions we made. Despite our efforts to augment state reported case counts with WWS data, it is likely that case count data overrepresent certain demographics within the study population. Individuals affiliated with Ohio State University and hospital workers had the highest access to COVID-19 testing during this study period due to robust free testing programs. These individuals may have certain demographic traits that are not representative of the entire study population. For example, the socioeconomic status of this subgroup may be different than that of the average individual in the study site. Importantly, both the infection distribution and perceived access to testing for COVID-19 is associated with socioeconomic status and other social determinants of health [35,67,68]. Since we assumed a homogeneous population with uniform contact rates, we may potentially overextend our findings on transmission variation and immune responses to communities within the sewershed not affiliated with a hospital or the university. We also assumed a uniform vaccine with a uniform effectiveness in our study. However, monovalent COVID-19 vaccinations by three different manufacturers were available to the study population: Moderna, Johnson & Johnson, and Pfizer-BioNTech. These vaccines likely vary in efficacy and protection against reinfection [64,69] and we do not capture the nuance of these potential differences. Finally, we recognize that immune-specific assays may be a more precise method to measure waning immunity, especially since antibody levels can vary greatly among individuals [70]. However, the benefit of using our methodology to estimate waning immunity is that it is non-invasive and represents the whole population, which can be especially useful for public health planning.

In conclusion, we demonstrate how the large amounts of data collected early in the pandemic can be used to reconstruct the transmission of a novel pathogen and capture changes in host immune responses among an initially susceptible human population. Transmission of SARS-CoV-2 did not display the consistent seasonal patterns observed in other acute, immunizing diseases in the first two years of its circulation. However, like other respiratory viruses, peaks in transmission may still have been attributed to changes in human behaviors, such as aggregation during school terms, and switches in the circulating viral strains. Our models highlight that immune protection is lost over time and that infection-induced and vaccine-induced immunity wane at similar rates. Elucidating these transmission dynamics and immune responses among the population as a whole can inform more effective vaccination strategies and schedules in the future. The approach used in this study can be easily applied other viral infectious disease models using large data from human cases and wastewater surveillance.

## Supporting information

S1 File Supplementary material. This file includes additional information on the study population (Text 1), disease data (Text 2 and Table 3), model equations (Text 3), parameter estimation (Text 4), and model parameter values (Table 1 and Table 2).
(PDF)
