# Supplementary material for: Quantifying transmission and immunity dynamics of multiple SARS-CoV-2 variants using models and epidemic data from a highly populated area
Source: PLoS One. 2025 Jul 16;20(7):e0327817. doi: 10.1371/journal.pone.0327817 (PMC12266459; doi:10.1371/journal.pone.0327817)
Supplement: S1 File — This file includes additional information on the study population (Text 1), disease data (Text 2 and Table 3), model equations (Text 3), parameter estimation (Text 4), and model parameter values (Table 1 and Table 2). (PDF) [file pone.0327817.s001.pdf]

Supplementary File for Quantifying transmission and immunity dynamics of multiple SARS-CoV-2 variants using models and epidemic data from a highly populated area

1. Study population and data

1.1 Scaling factor

Franklin county is the most populous county in Ohio with 1,321,414 residents (United States Census Bureau, 2022). Assuming a uniform population density throughout Franklin County, we calculated that the Jackson Pike sewershed serves 49% of Franklin County residents. We used this proportion as a scaling factor for all county-level birth, mortality, vaccination, and disease data.

1.2 Birth and mortality data

To account for population dynamics during the two years of our study, we included births and deaths in each of our models. Data on live births were retrieved from the Ohio Department of Health (ODH) Public Health Information Warehouse (Ohio Department of Health, 2023). These data contain counts of residents in Franklin County who had a live birth per month per year. Because complete birth data from 2021 and 2022 were not yet available, we averaged birth counts for each month from 2016 to 2020. We then multiplied average monthly births by the Jackson Pike scaling factor described above to find the number of births, per month, in the study area. We divided each scaled monthly average by the number of weeks in the respective month to get counts of births for each week. We used these weekly values in our model as the time-varying count "Number of births" (b_t).

Data on mortality attributable to all causes other than COVID-19 were also retrieved from the ODH Public Health Information Warehouse (Ohio Department of Health, 2023). These data contain the number of deaths in Franklin County per month, per year. As with birth data, complete death data from 2021 and 2022 were not yet available, so we averaged death counts for each month from 2016 to 2020 and multiplied counts by the Jackson Pike scaling factor to quantify deaths in the study area. We subsequently converted this value into the average proportion of the Jackson Pike population dying each week due to causes other than COVID-19 and used this in our models as "Proportion dying attributable to all causes except COVID-19" ($\mu_{1,t}$).

COVID-19 specific mortality data were retrieved from the ODH COVID-19 dashboard (Ohio Department of Health, 2022a). Since these mortality data were reported daily by county, we multiplied counts reported from Franklin County by the Jackson Pike scaling factor to quantify COVID-19 deaths in the study area. We aggregated daily data into weekly counts and then divided by the study population size to calculate the proportion of the Jackson Pike population dying due to COVID-19 each week and used this in our models as "Proportion of infected individuals dying due to COVID-19" ($\mu_{2,t}$).

2. Disease and vaccination data

2.1 Disease data

Daily COVID-19 case count data: These data daily contain counts of positive tests reported to the state for residents that live inside the service boundaries of the Jackson Pike sewershed and the date of symptom onset or reporting. We aggregated these data by week (beginning on Sunday).

Wastewater surveillance data: We estimated the prevalence, or the total number of infected individuals in a 24-hour period, from wastewater data using the simplified equation described in McMahan et. al (2021) (Equation 1). Let J represent the number of infected individuals in the population at time t, Q represent the flow rate at time t, V represent the number of gene copies per liter at time t, A represent the grams of

feces produced by individual per day, and B represent the maximum number of gene copies shed through feces (McMahan et al., 2021).

$$J_t = \frac{Q_t * V_t}{A * B} \quad (1)$$

Since the Jackson Pike Treatment plant is sampled twice per week (24-hr composite samples) on Tuesdays and Sundays, we used the equation to yield an estimate of the number of infected individuals on those days. We then averaged the two daily values, which gave an average prevalence for the week. Because COVID-19 has an infectious period 7 days (Byrne et al., 2020), we can align this averaged prevalence to weekly incidence. We assume that those who are infected during week t are no longer infected during week $t + 1$, and all infected individuals detected during $t + 1$ are newly infected cases. We set A and B as the same values used in the study by McMahan et al. 2021. We then compared our weekly estimates of SARS-CoV-2 infections from wastewater data to the number of cases reported to the state. We used this wastewater-derived weekly estimated number of infected individuals as the lower bound for the magnitude of cases underreported parameter in our models.

2.2 Vaccination efficacy
We used the clinal trial efficacy data from the BNT162b2 monovalent vaccine to base this assumption; the vaccine indicates a 94.6% efficacy as soon as 7 days after completing the primary series and a 94.7% efficacy as soon as 7 days after completing the first booster (Moreira et al., 2022; Polack et al., 2020). However, studies have estimated a lower vaccine effectiveness under real-world settings (Bloomfield et al., 2023; Thompson, 2021) While neither of these is a direct measure of the proportion that mounts immunity, we use this to guide our assumption that only 90% of those who receive the vaccine move into the vaccinated category.

3. Models of transmission and immunity

Full list of model equations:

$$\lambda_t = \beta_t I_t$$

$$S_{t+1} = S_t + \omega R_t - \lambda_t S_t + \nu_2 V_t - \varepsilon \nu_{1,t} S_t - \mu_{1,t} S_t + b_t$$

$$E_{t+1} = E_t + \lambda_t S_t - \sigma E_t$$

$$I_{t+1} = I_t + \sigma E_t - \gamma I_t - \mu_{2,t} I_t$$

$$R_{t+1} = R_t + \gamma I_t - \omega R_t - \mu_{1,t} R_t$$

$$V_{t+1} = V_t + \varepsilon \nu_{1,t} S_t - \nu_2 V_t - \mu_{1,t} V_t$$

4. *Model selection, implementation, and computing*

4.1 *Parameter estimation*
We estimated a monthly parameter value for the transmission coefficient (β_t) by constraining weekly parameter estimates such that four or five weekly successive values were estimated as the same number. We chose which four or five weekly successive parameter values were estimated as the same number based on monthly calendars. We performed the estimation for each parameter (β_t, ω, and ν_2) ten times,

iteratively, using estimated values from the previous optimization as starting points for the next optimization.

Table 1. Model parameter values.

Parameter	Definition	Value	Source
γ	Inverse of recovery period	0.7 $week^{-1}$	(Centers for Disease Control and Prevention, 2023)
σ	Inverse of latency period	1 $week^{-1}$	Estimated from (Xin et al., 2022)
ε	Proportion who mounts protective immunity from receiving vaccine	0.90	Assumed from (Moreira et al., 2022; Polack et al., 2020)
b_t	Number of births	Time-varying; Table 2	(Ohio Department of Health, 2023)
$\mu_{1,t}$	Proportion dying attributable to all causes except COVID-19	Time-varying; Table 2	(Ohio Department of Health, 2023)
$\mu_{2,t}$	Proportion of infected individuals dying due to COVID-19	Time-varying; Table 2	(Ohio Department of Health, 2022a)
$\nu_{1,t}$	Proportion receiving vaccination	Time-varying; Table 2	(Ohio Department of Health, 2022a)

Table 2. Full list of time varying parameter values.

Week Number	Date	Flow rate (liter/day) **(Q)**	Gene copies per liter **(V)**	Number of births **(b)**	Proportion dying attributable to all causes except COVID-19 **(μ_1)**	Proportion of infected individuals dying due to COVID-19 **(μ_2)**	Proportion receiving vaccination **(ν_1)**
1	1/26/20	-	-	183	1.75E-04	0	0
2	2/2/20	-	-	169	1.57E-04	0	0
3	2/9/20	-	-	169	1.57E-04	0	0
4	2/16/20	-	-	169	1.57E-04	0	0
5	2/23/20	-	-	169	1.57E-04	0	0
6	3/1/20	-	-	183	1.67E-04	0	0
7	3/8/20	-	-	183	1.67E-04	0	0

8	3/15/20	-	-	183	1.67E-04	1.51E-06	0
9	3/22/20	-	-	183	1.67E-04	2.27E-06	0
10	3/29/20	-	-	183	1.67E-04	5.30E-06	0
11	4/5/20	-	-	182	1.62E-04	1.06E-05	0
12	4/12/20	-	-	182	1.62E-04	1.06E-05	0
13	4/19/20	-	-	182	1.62E-04	1.66E-05	0
14	4/26/20	-	-	182	1.62E-04	2.04E-05	0
15	5/3/20	-	-	190	1.65E-04	2.72E-05	0
16	5/10/20	-	-	190	1.65E-04	3.25E-05	0
17	5/17/20	-	-	190	1.65E-04	3.33E-05	0
18	5/24/20	-	-	190	1.65E-04	2.12E-05	0
19	5/31/20	-	-	190	1.65E-04	2.50E-05	0
20	6/7/20	-	-	188	1.53E-04	1.82E-05	0
21	6/14/20	-	-	188	1.53E-04	1.82E-05	0
22	6/21/20	-	-	188	1.53E-04	1.66E-05	0
23	6/28/20	-	-	188	1.53E-04	1.36E-05	0
24	7/5/20	-	-	198	1.56E-04	1.36E-05	0
25	7/12/20	-	-	198	1.56E-04	1.36E-05	0
26	7/19/20	2.4E+08	785	198	1.56E-04	1.14E-05	0
27	7/26/20	2.5E+08	316	198	1.56E-04	7.57E-06	0
28	8/2/20	2.74E+08	1469	203	1.54E-04	1.36E-05	0
29	8/9/20	2.55E+08	1716	203	1.53E-04	1.14E-05	0
30	8/16/20	2.63E+08	1866	203	1.53E-04	1.06E-05	0
31	8/23/20	2.52E+08	2166.5	203	1.54E-04	1.21E-05	0
32	8/30/20	2.5E+08	2587	203	1.54E-04	3.78E-06	0
33	9/6/20	4.78E+08	5366	195	1.50E-04	1.14E-05	0
34	9/13/20	3.02E+08	5775.5	195	1.50E-04	6.05E-06	0
35	9/20/20	2.46E+08	7102.5	195	1.50E-04	9.08E-06	0
36	9/27/20	2.68E+08	6989	195	1.50E-04	9.08E-06	0
37	10/4/20	2.57E+08	12200	190	1.65E-04	9.08E-06	0
38	10/11/20	2.73E+08	8547.5	190	1.65E-04	2.27E-06	0
39	10/18/20	3.24E+08	9790	190	1.65E-04	1.06E-05	0
40	10/25/20	2.58E+08	29796	190	1.65E-04	8.32E-06	0
41	11/1/20	2.66E+08	34179	181	1.66E-04	2.04E-05	0
42	11/8/20	3.21E+08	19750	181	1.66E-04	1.89E-05	0
43	11/15/20	2.54E+08	41645.5	181	1.66E-04	3.03E-05	0

44	11/22/20	3.27E+08	54741	181	1.66E-04	4.77E-05	0
45	11/29/20	3.31E+08	81257	181	1.66E-04	4.84E-05	0
46	12/6/20	2.77E+08	80511	183	1.87E-04	5.52E-05	0
47	12/13/20	2.12E+08	59458	183	1.87E-04	5.68E-05	7.57E-07
48	12/20/20	3.21E+08	59900	183	1.87E-04	5.07E-05	3.03E-06
49	12/27/20	2.79E+08	42041	183	1.87E-04	4.16E-05	3.41E-05
50	1/3/21	4.32E+08	86745.5	183	1.75E-04	3.56E-05	9.66E-04
51	1/10/21	2.04E+08	51933.5	183	1.75E-04	4.24E-05	1.84E-03
52	1/17/21	1.84E+08	37317	183	1.75E-04	4.31E-05	4.66E-03
53	1/24/21	3.39E+08	17731.5	183	1.75E-04	2.42E-05	1.03E-02
54	1/31/21	3.26E+08	19370	183	1.75E-04	2.80E-05	1.35E-02
55	2/7/21	3.16E+08	5022	169	1.57E-04	2.27E-05	1.62E-02
56	2/14/21	2.81E+08	17285	169	1.57E-04	7.57E-06	1.46E-02
57	2/21/21	3.34E+08	9760.5	169	1.57E-04	1.21E-05	2.33E-02
58	2/28/21	4.58E+08	8496	169	1.57E-04	1.36E-05	1.59E-02
59	3/7/21	2.9E+08	13652.5	183	1.67E-04	7.57E-06	1.71E-02
60	3/14/21	2.93E+08	19611.5	183	1.67E-04	3.03E-06	1.37E-02
61	3/21/21	3.27E+08	16683.5	183	1.67E-04	5.30E-06	2.00E-02
62	3/28/21	3.05E+08	22026	183	1.67E-04	4.54E-06	2.85E-02
63	4/4/21	2.89E+08	10492	182	1.62E-04	3.78E-06	4.26E-02
64	4/11/21	3.24E+08	12942	182	1.62E-04	6.05E-06	3.87E-02
65	4/18/21	2.61E+08	19055.5	182	1.62E-04	1.21E-05	3.97E-02
66	4/25/21	2.64E+08	12152	182	1.62E-04	5.30E-06	4.18E-02
67	5/2/21	3.84E+08	4529	190	1.65E-04	8.32E-06	3.51E-02
68	5/9/21	5.88E+08	791	190	1.65E-04	9.08E-06	2.48E-02
69	5/16/21	2.87E+08	6386	190	1.65E-04	7.57E-06	1.67E-02
70	5/23/21	2.72E+08	2428.5	190	1.65E-04	7.57E-06	1.16E-02
71	5/30/21	2.54E+08	493	190	1.65E-04	5.30E-06	1.24E-02
72	6/6/21	2.85E+08	337	188	1.53E-04	5.30E-06	1.67E-02
73	6/13/21	2.58E+08	464	188	1.53E-04	4.54E-06	1.20E-02
74	6/20/21	2.68E+08	526	188	1.53E-04	3.78E-06	7.86E-03
75	6/27/21	2.32E+08	278	188	1.53E-04	1.51E-06	6.12E-03
76	7/4/21	2.66E+08	1190	198	1.56E-04	2.27E-06	5.09E-03
77	7/11/21	4.11E+08	1283	198	1.56E-04	1.51E-06	4.54E-03
78	7/18/21	3.23E+08	8520	198	1.56E-04	1.51E-06	4.08E-03
79	7/25/21	2.57E+08	2767.5	198	1.56E-04	3.78E-06	4.04E-03

80	8/1/21	2.81E+08	4111.5	203	1.54E-04	2.27E-06	4.04E-03
81	8/8/21	2.85E+08	10550	203	1.54E-04	4.54E-06	5.02E-03
82	8/15/21	3.97E+08	7457.5	203	1.54E-04	6.05E-06	6.89E-03
83	8/22/21	2.59E+08	8320	203	1.54E-04	1.21E-05	7.03E-03
84	8/29/21	3.14E+08	8303	203	1.54E-04	1.59E-05	7.35E-03
85	9/5/21	2.66E+08	2541	195	1.50E-04	2.19E-05	6.07E-03
86	9/12/21	2.63E+08	8550	195	1.50E-04	2.42E-05	5.98E-03
87	9/19/21	2.75E+08	6403.5	195	1.50E-04	2.19E-05	7.04E-03
88	9/26/21	2.69E+08	10621	195	1.50E-04	2.27E-05	1.51E-02
89	10/3/21	2.75E+08	5834	190	1.65E-04	2.80E-05	1.41E-02
90	10/10/21	2.5E+08	3892	190	1.65E-04	2.19E-05	1.33E-02
91	10/17/21	2.64E+08	7020.5	190	1.65E-04	3.33E-05	1.37E-02
92	10/24/21	3.27E+08	10550	190	1.65E-04	2.04E-05	2.23E-02
93	10/31/21	2.89E+08	5450	190	1.65E-04	1.66E-05	2.39E-02
94	11/7/21	2.62E+08	29095	181	1.66E-04	1.89E-05	2.03E-02
95	11/14/21	2.83E+08	28025	181	1.66E-04	1.51E-05	2.28E-02
96	11/21/21	3.18E+08	24750	181	1.66E-04	1.21E-05	1.99E-02
97	11/28/21	2.72E+08	19505	181	1.66E-04	2.04E-05	3.26E-02
98	12/5/21	3.8E+08	14942	183	1.87E-04	3.56E-05	3.30E-02
99	12/12/21	3.21E+08	28877.5	183	1.87E-04	2.72E-05	3.01E-02
100	12/19/21	3.04E+08	56468	183	1.87E-04	4.62E-05	2.26E-02
101	12/26/21	3.29E+08	95254	183	1.87E-04	4.46E-05	2.02E-02
102	1/2/22	5.02E+08	54312.5	183	1.75E-04	4.54E-05	2.31E-02
103	1/9/22	4.03E+08	38010	183	1.75E-04	6.43E-05	1.82E-02
104	1/16/22	2.88E+08	61524	183	1.75E-04	6.28E-05	1.31E-02
105	1/23/22	2.82E+08	47829	183	1.75E-04	5.30E-05	9.39E-03
106	1/30/22	2.73E+08	32698	183	1.75E-04	3.56E-05	5.74E-03
107	2/6/22	3.24E+08	12873	169	1.57E-04	3.03E-05	6.01E-03
108	2/13/22	3.32E+08	9953.5	169	1.57E-04	2.27E-05	4.74E-03
109	2/20/22	5.49E+08	3985	169	1.57E-04	1.14E-05	3.75E-03
110	2/27/22	3.94E+08	3102	169	1.57E-04	1.06E-05	3.27E-03
111	3/6/22	4.58E+08	2553.5	183	1.67E-04	4.54E-06	2.91E-03
112	3/13/22	3.16E+08	3203	183	1.67E-04	6.81E-06	2.60E-03
113	3/20/22	3.09E+08	2862.5	183	1.67E-04	4.54E-06	2.37E-03
114	3/27/22	2.81E+08	4586	183	1.67E-04	3.03E-06	2.22E-03
115	4/3/22	2.72E+08	7695.5	183	1.62E-04	3.03E-06	2.00E-03

116	4/10/22	2.95E+08	6119	183	1.62E-04	3.03E-06	1.96E-03
117	4/17/22	3.54E+08	8419	183	1.62E-04	1.51E-06	1.64E-03
118	4/24/22	3.28E+08	11201	183	1.62E-04	2.27E-06	1.67E-03
119	5/1/22	3.39E+08	11224	190	1.65E-04	7.57E-07	1.62E-03
120	5/8/22	4.27E+08	11103	190	1.65E-04	7.57E-07	1.61E-03
121	5/15/22	3.46E+08	11295	190	1.65E-04	1.51E-06	1.82E-03
122	5/22/22	3.4E+08	5708	190	1.65E-04	7.57E-07	2.12E-03
123	5/29/22	3.11E+08	13680	190	1.65E-04	2.27E-06	1.67E-03
124	6/5/22	3.76E+08	5296	188	1.53E-04	4.54E-06	1.77E-03
125	6/12/22	3.15E+08	2784.5	188	1.53E-04	3.03E-06	1.44E-03
126	6/19/22	2.87E+08	23160	188	1.53E-04	4.54E-06	1.43E-03
127	6/26/22	2.68E+08	7742.5	188	1.53E-04	3.03E-06	1.42E-03
128	7/3/22	-	-	198	1.56E-04	3.78E-06	1.05E-03
129	7/10/22	3.08E+08	17040	198	1.56E-04	4.54E-06	1.64E-03
130	7/17/22	4.64E+08	18615	198	1.56E-04	2.27E-06	2.16E-03
131	7/24/22	2.88E+08	19009	198	1.56E-04	6.05E-06	2.25E-03
132	7/31/22	2.72E+08	26663.5	198	1.56E-04	4.54E-06	1.97E-03
133	8/7/22	2.93E+08	23660.5	203	1.54E-04	7.57E-06	2.01E-03
134	8/14/22	2.52E+08	55108	203	1.54E-04	2.27E-06	1.59E-03
135	8/21/22	3.81E+08	24347.5	203	1.54E-04	3.78E-06	1.19E-03

Table 3. Case data in Jackson Pike Sewershed by week. Number of cases reported by week (Ohio Department of Health, 2022b).

Week Number	Date	Case count
1	1/26/20	2
2	2/2/20	1
3	2/9/20	2
4	2/16/20	0
5	2/23/20	8
6	3/1/20	24
7	3/8/20	52
8	3/15/20	155
9	3/22/20	112
10	3/29/20	119
11	4/5/20	117

12	4/12/20	165
13	4/19/20	237
14	4/26/20	398
15	5/3/20	231
16	5/10/20	318
17	5/17/20	265
18	5/24/20	217
19	5/31/20	225
20	6/7/20	248
21	6/14/20	299
22	6/21/20	514
23	6/28/20	732
24	7/5/20	1116
25	7/12/20	907
26	7/19/20	611
27	7/26/20	503
28	8/2/20	454
29	8/9/20	413
30	8/16/20	431
31	8/23/20	880
32	8/30/20	1308
33	9/6/20	917
34	9/13/20	564
35	9/20/20	568
36	9/27/20	556
37	10/4/20	684
38	10/11/20	774
39	10/18/20	920
40	10/25/20	1349
41	11/1/20	2192
42	11/8/20	2966
43	11/15/20	3036
44	11/22/20	2777
45	11/29/20	3175
46	12/6/20	2751
47	12/13/20	2560
48	12/20/20	2307
49	12/27/20	2598
50	1/3/21	2744

51	1/10/21	2188
52	1/17/21	1707
53	1/24/21	1247
54	1/31/21	1001
55	2/7/21	810
56	2/14/21	708
57	2/21/21	634
58	2/28/21	677
59	3/7/21	626
60	3/14/21	582
61	3/21/21	769
62	3/28/21	752
63	4/4/21	786
64	4/11/21	721
65	4/18/21	527
66	4/25/21	431
67	5/2/21	390
68	5/9/21	296
69	5/16/21	265
70	5/23/21	213
71	5/30/21	115
72	6/6/21	110
73	6/13/21	81
74	6/20/21	68
75	6/27/21	73
76	7/4/21	106
77	7/11/21	173
78	7/18/21	272
79	7/25/21	431
80	8/1/21	738
81	8/8/21	934
82	8/15/21	1051
83	8/22/21	1325
84	8/29/21	1672
85	9/5/21	1640
86	9/12/21	1841
87	9/19/21	1516
88	9/26/21	1377
89	10/3/21	1219

90	10/10/21	975
91	10/17/21	901
92	10/24/21	847
93	10/31/21	983
94	11/7/21	1146
95	11/14/21	1293
96	11/21/21	1250
97	11/28/21	2016
98	12/5/21	1731
99	12/12/21	2272
100	12/19/21	4561
101	12/26/21	8884
102	1/2/22	11088
103	1/9/22	9028
104	1/16/22	5654
105	1/23/22	3362
106	1/30/22	1807
107	2/6/22	1011
108	2/13/22	637
109	2/20/22	395
110	2/27/22	277
111	3/6/22	205
112	3/13/22	196
113	3/20/22	268
114	3/27/22	330
115	4/3/22	385
116	4/10/22	532
117	4/17/22	638
118	4/24/22	908
119	5/1/22	1126
120	5/8/22	1448
121	5/15/22	1433
122	5/22/22	1340
123	5/29/22	1218
124	6/5/22	1150
125	6/12/22	955
126	6/19/22	1130
127	6/26/22	1197
128	7/3/22	1190

129	7/10/22	1242
130	7/17/22	1358
131	7/24/22	1266
132	7/31/22	1142
133	8/7/22	1122
134	8/14/22	898
135	8/21/22	978

References:

Bloomfield, L.E., Ngeh, S., Cadby, G., Hutcheon, K., Effler, P.V., 2023. SARS-CoV-2 Vaccine Effectiveness against Omicron Variant in Infection-Naive Population, Australia, 2022 - Volume 29, Number 6—June 2023 - Emerging Infectious Diseases journal - CDC. https://doi.org/10.3201/eid2906.230130

Byrne, A.W., McEvoy, D., Collins, A.B., Hunt, K., Casey, M., Barber, A., Butler, F., Griffin, J., Lane, E.A., McAloon, C., O'Brien, K., Wall, P., Walsh, K.A., More, S.J., 2020. Inferred duration of infectious period of SARS-CoV-2: rapid scoping review and analysis of available evidence for asymptomatic and symptomatic COVID-19 cases. BMJ Open 10, e039856. https://doi.org/10.1136/bmjopen-2020-039856

Centers for Disease Control and Prevention, 2023. Isolation [WWW Document]. Centers for Disease Control and Prevention. URL https://www.cdc.gov/coronavirus/2019-ncov/your-health/isolation.html (accessed 3.27.23).

McMahan, C.S., Self, S., Rennert, L., Kalbaugh, C., Kriebel, D., Graves, D., Colby, C., Deaver, J.A., Popat, S.C., Karanfil, T., Freedman, D.L., 2021. COVID-19 wastewater epidemiology: a model to estimate infected populations. The Lancet Planetary Health 5, e874–e881. https://doi.org/10.1016/S2542-5196(21)00230-8

Moreira, E.D., Kitchin, N., Xu, X., Dychter, S.S., Lockhart, S., Gurtman, A., Perez, J.L., Zerbini, C., Dever, M.E., Jennings, T.W., Brandon, D.M., Cannon, K.D., Koren, M.J., Denham, D.S., Berhe, M., Fitz-Patrick, D., Hammitt, L.L., Klein, N.P., Nell, H., Keep, G., Wang, X., Koury, K., Swanson, K.A., Cooper, D., Lu, C., Türeci, Ö., Lagkadinou, E., Tresnan, D.B., Dormitzer, P.R., Şahin, U., Gruber, W.C., Jansen, K.U., 2022. Safety and Efficacy of a Third Dose of BNT162b2 Covid-19 Vaccine. New England Journal of Medicine 386, 1910–1921. https://doi.org/10.1056/NEJMoa2200674

Ohio Department of Health, 2023. Ohio Public Health Information Warehouse - Public: Dataset Catalog [WWW Document]. URL https://publicapps.odh.ohio.gov/EDW/DataCatalog (accessed 3.27.23).

Ohio Department of Health, 2022a. COVID-19 Dashboard [WWW Document]. URL https://public.tableau.com/views/OverviewDashboard_15852499073250/DashboardOverview_1?:embed=y&:showVizHome=no&:host_url=https%3A%2F%2Fpublic.tableau.com%2F&:embed_code_version=3&:tabs=no&:toolbar=no&:showAppBanner=false&iframeSizedToWindow=true&:loadOrderID=0 (accessed 4.11.22).

Ohio Department of Health, 2022b. Ohio Coronavirus Wastwater Monitoring Network [WWW Document]. URL https://coronavirus.ohio.gov/dashboards/other-resources/wastewater (accessed 4.11.22).

Polack, F.P., Thomas, S.J., Kitchin, N., Absalon, J., Gurtman, A., Lockhart, S., Perez, J.L., Pérez Marc, G., Moreira, E.D., Zerbini, C., Bailey, R., Swanson, K.A., Roychoudhury, S., Koury, K., Li, P., Kalina, W.V., Cooper, D., Frenck, R.W., Hammitt, L.L., Türeci, Ö., Nell, H., Schaefer, A., Ünal, S., Tresnan, D.B., Mather, S., Dormitzer, P.R., Şahin, U., Jansen, K.U., Gruber, W.C., 2020. Safety and Efficacy of the BNT162b2 mRNA Covid-19 Vaccine. New England Journal of Medicine 383, 2603–2615. https://doi.org/10.1056/NEJMoa2034577

Thompson, M.G., 2021. Interim Estimates of Vaccine Effectiveness of BNT162b2 and mRNA-1273 COVID-19 Vaccines in Preventing SARS-CoV-2 Infection Among Health Care Personnel, First Responders, and Other Essential and Frontline Workers — Eight U.S. Locations, December 2020–March 2021. MMWR Morb Mortal Wkly Rep 70. https://doi.org/10.15585/mmwr.mm7013e3

United States Census Bureau, 2022. U.S. Census Bureau QuickFacts: Franklin County, Ohio [WWW Document]. URL https://www.census.gov/quickfacts/franklincountyohio (accessed 3.27.23).

Xin, H., Li, Y., Wu, P., Li, Zhili, Lau, E.H.Y., Qin, Y., Wang, L., Cowling, B.J., Tsang, T.K., Li, Zhongjie, 2022. Estimating the Latent Period of Coronavirus Disease 2019 (COVID-19). Clinical Infectious Diseases 74, 1678–1681. https://doi.org/10.1093/cid/ciab746
